# Visualizing genomic information across chromosomes with PhenoGram

**DOI:** 10.1186/1756-0381-6-18

**Published:** 2013-10-16

**Authors:** Daniel Wolfe, Scott Dudek, Marylyn D Ritchie, Sarah A Pendergrass

**Affiliations:** 1 Center for Systems Genomics, Department of Biochemistry and Molecular Biology, Eberly College of Science, The Huck Institutes of the Life Sciences, The Pennsylvania State University, 512 Wartik Laboratory, University Park, PA 16802, USA

**Keywords:** Data visualization, Bioinformatics, Genome-wide association study, GWAS, Copy-number variants, CNV, SNP, Ideogram

## Abstract

**Background:**

With the abundance of information and analysis results being collected for genetic loci, user-friendly and flexible data visualization approaches can inform and improve the analysis and dissemination of these data. A chromosomal ideogram is an idealized graphic representation of chromosomes. Ideograms can be combined with overlaid points, lines, and/or shapes, to provide summary information from studies of various kinds, such as genome-wide association studies or phenome-wide association studies, coupled with genomic location information. To facilitate visualizing varied data in multiple ways using ideograms, we have developed a flexible software tool called PhenoGram which exists as a web-based tool and also a command-line program.

**Results:**

With PhenoGram researchers can create chomosomal ideograms annotated with lines in color at specific base-pair locations, or colored base-pair to base-pair regions, with or without other annotation. PhenoGram allows for annotation of chromosomal locations and/or regions with shapes in different colors, gene identifiers, or other text. PhenoGram also allows for creation of plots showing expanded chromosomal locations, providing a way to show results for specific chromosomal regions in greater detail. We have now used PhenoGram to produce a variety of different plots, and provide these as examples herein. These plots include visualization of the genomic coverage of SNPs from a genotyping array, highlighting the chromosomal coverage of imputed SNPs, copy-number variation region coverage, as well as plots similar to the NHGRI GWA Catalog of genome-wide association results.

**Conclusions:**

PhenoGram is a versatile, user-friendly software tool fostering the exploration and sharing of genomic information. Through visualization of data, researchers can both explore and share complex results, facilitating a greater understanding of these data.

## Background

As the types and amount of genomic data being collected continue to increase, so does the need for tools to visualize, analyze, and share these data. One useful data visualization approach for genomic results is the use of chromosomal ideograms. An ideogram is a graphical representation of chromosomes, and these plots have been used with the addition of overlaid points, lines, and shapes to provide summary information of various kinds coupled with genomic location information [1,2]. For example, the National Human Genome Research Institute (NHGRI) Genome-Wide Association Study (GWAS) Catalog has plotted the results of multiple genome-wide association studies using ideograms, highlighting genomic regions and a range of associated phenotypes for current published GWAS (http://www.genome.gov/gwastudies/) [3].

Any –omic data that can be represented by chromosomal base pair locations or regions can also be plotted with ideograms. Genotyping array coverage information, single nucleotide polymorphism (SNP) imputation results, and the results of association studies with multiple phenotypes such as phenome-wide associations studies (PheWAS) [4,5], are examples of other types of data that can benefit from the broad perspective offered by visualizing data with a chromosomal ideogram. The software PhenoGram has been developed to meet the need for an accessible tool that can allow researchers to both better understand complex data and easily disseminate the results.

PhenoGram was initially conceived as a method to highlight SNP-phenotype association results across the genome through the use of color-coded circles corresponding to various phenotypes, linked by lines to genomic locations, similar to the aforementioned NHGRI GWAS Catalog plots. We subsequently expanded the PhenoGram feature set, providing more options for other types of plots. Via the command line or on the web using a graphical interface, researchers can supply different types of information along with base-pair or region data that plotted onto an ideogram according to the researcher’s preferences. Resulting PhenoGram plots can be downloaded as 1200 dots per inch (DPI) lossless PNG images that are publication ready.

For example, researchers can annotate chromosomal locations or biologically relevant regions to indicate traits associated with specific positions, and can choose different shapes to highlight ancestry or another study attribute related to specific data points. The use of PhenoGram is not limited to association results, as it can be used to plot and annotate chromosome regions across an ideogram without phenotype information. PhenoGram offers a complete genomic picture. Data that relates gene loci, phenotypes, or other attributes to genome location can be complex, and summarizing such data with visualization methods can be important for better understanding results.

### Implementation

**Figure 1:**
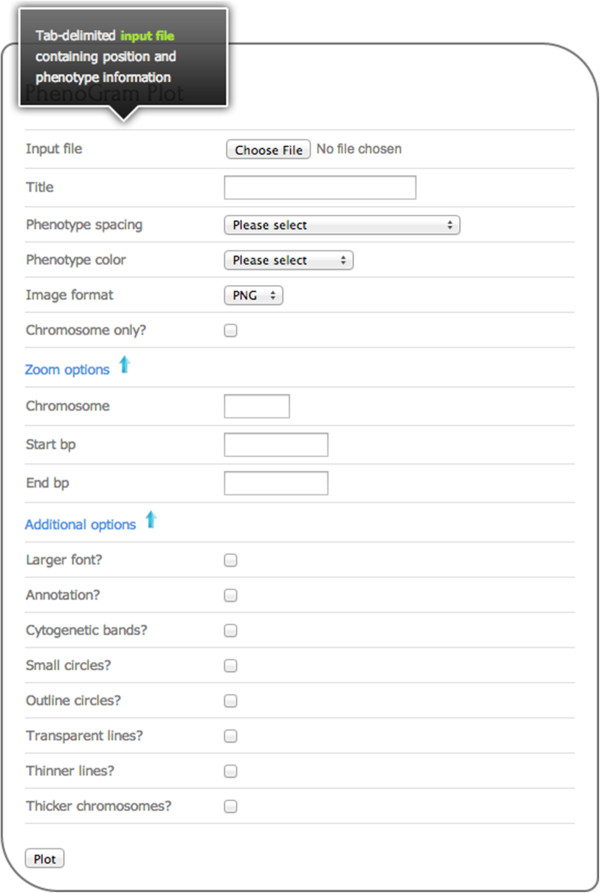
**Screen capture of the PhenoGram web-interface.** The researcher will upload an input file, provide a title of the resultant figure, and then choose other options. Scrolling over each item, such as “Input File” will provide information on what the line means, as well as a link to example files that can be used with Phenogram when relevant. For example, scrolling over “Input file” indicates a tab-delimited input file is necessary, and provides a link to an example file that can be used with PhenoGram.

PhenoGram was developed in Ruby, using the RMagick graphics library. The software can be downloaded for use at the command line. The software can also be used via a web-based graphical user interface without a need for downloading the software, and a screen capture of the web interface is shown in Figure 1. Both the web-based graphical user interface as well as the stand-alone software are available at: http://visualization.ritchielab.psu.edu. An example file is available at that site for trying out PhenoGram with the web-based graphical user interface.

There are multiple options that can be used to create various plots, and Table 1 shows the complete list of command line arguments, which are also available on the web interface. A single, tab-delimited input file is required to produce a PhenoGram plot. At a minimum, the input file must contain columns to identify the chromosome, and the base-pair position or base-pair to base-pair region to be plotted. Other columns such phenotype, annotation, ancestry or group, and position-color provide additional PhenoGram visualization options. Table 2 summarizes the formatting parameters of the input file.

**Table 1:** **PhenoGram plotting options and arguments (−*arg name) *** **for creating PhenoGram plots using pheno_gram.rb**

**Usage: pheno_gram.rb**	
-h --help	Show the help message and exit
-v --version	Show PhenoGram version
-i *input filename*	Filename of input configuration file
-o *output filename*	Filename of output plot
-t *title*	Main plot title (enclose in double quotes)
-f *image type*	Output image format (default is PNG); other options depend on ImageMagick installation
-p *phenotype spacing*	Determines standard, equal, or alternative algorithm
-c *color range*	Determines random, web, generator, group, or list algorithm
-z --high-res	Sets plot resolution to 1200 DPI
-C --chrom-only	Plot only chromosomes with position
-S --small-circle	Plot with smaller phenotype circles
-O --outline-circle	Plot phenotype circles with black outline
-Z *zoom location*	Zoom on chromosome (i.e. 7) or region (i.e. 7:10000–20000)
-a --include-annotation	Include annotation on plot
-T --trans-lines	Show more transparent lines on chromosomes
-n --thin-lines	Show thinner lines across chromosomes
-B --thick-boundary	Increase thickness of chromosome boundary
-F --big-font	Increase font size of phenotype labels
-x –shade-chromatin	Add shading to inaccessible or cytogenetic chromosome regions
-r *random seed*	Seed for random number generator (default is 7)

**Table 2:** PhenoGram input file formatting parameters

**Recognized column header**	**Required**	**Description**
CHR	Yes	Chromosome number
POS	Yes	Base-pair location of the SNP or starting location of a base-pair to base-pair region
PHENOTYPE	No	Name of the phenotype; required only when plotting phenotypes as colored shapes
END	No	Ending location of a base-pair to base-pair region
NOTE (or ANNOTATION)	No	Values in this column are shown on the plot position to the right of the chromosome; limited to 10 characters
ETHNICITY (or ANCESTRY)	No	Specifies ethnicity or ancestry for the associated position; accepts up to three unique values in this column
GROUP	No	Specifies a group identifier such that all phenotypes of the same identifier share a common color
POSCOLOR	No	Shades transverse lines on the line plot with a color; specified by an integer 0-7

The file must be tab delimited. PhenoGram will ignore any unrecognized headers.

## Results and discussion

To show the utility of PhenoGram, and the ways that multiple options can be combined for different types of plots, we describe here several example uses of this software. For the first set of examples, we have used a subset of data from the NHGRI GWAS Catalog to demonstrate some features of PhenoGram, highlighting some of the similarities and differences in our plots compared to the NHGRI GWAS Catalog plots. We chose this data because allowed us to represent multiple phenotypes across the genome and highlight other relationships in the data such as pleiotropy or ancestry. In addition, the GWAS Catalog data could be prepared as input to PhenoGram with a single database query and minimal data.

**Figure 2:**
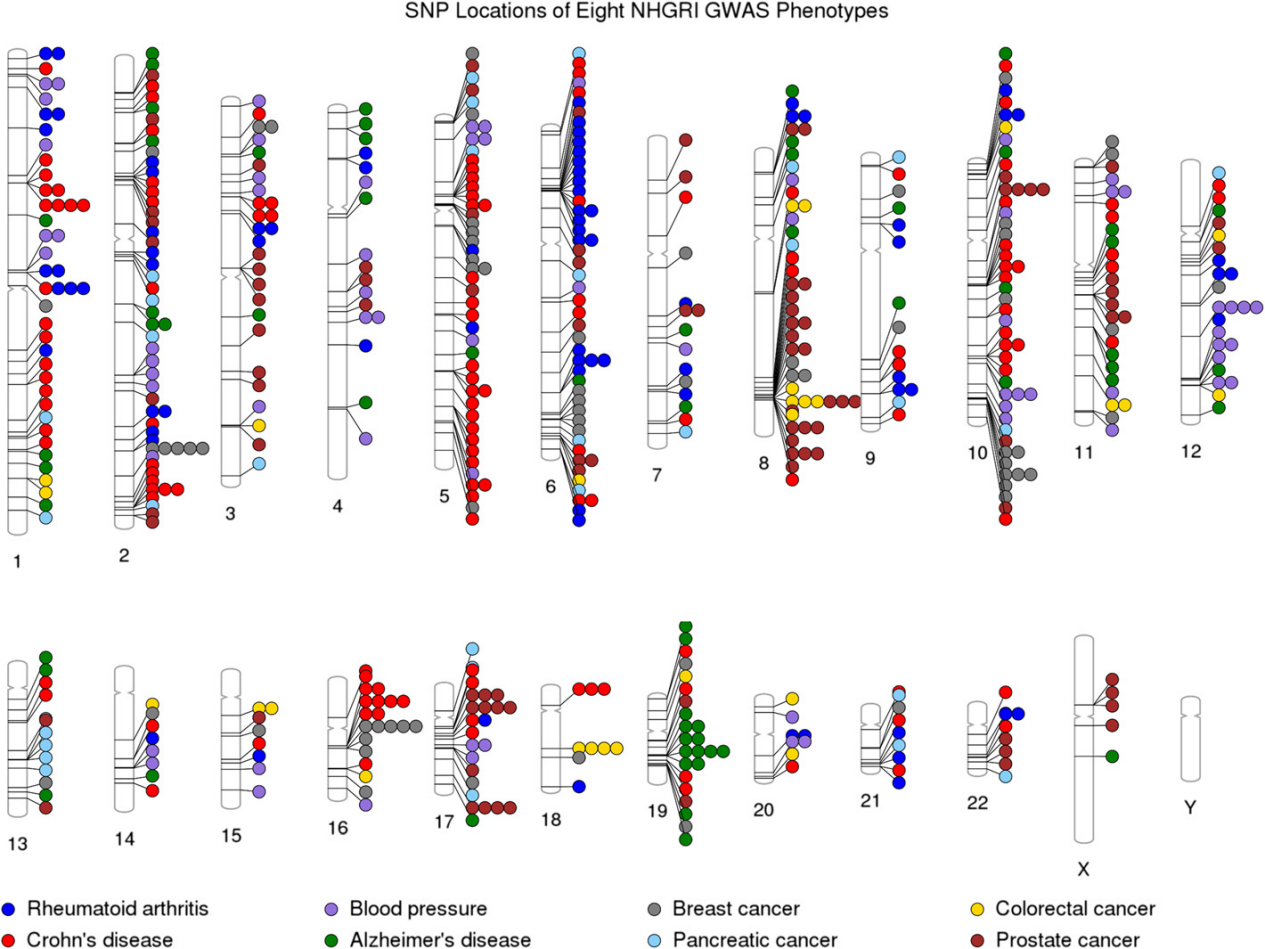
**Using PhenoGram to plot the NHGRI GWA catalog association results for eight phenotypes.** An Ideogram of all 22 chromosomes is plotted, along with the X and Y chromosomes. Lines are plotted on the chromosomes corresponding to the base-pair location of each SNP, and the line connects to colored circles representing the phenotype(s) associated with that SNP.

Here, we chose a subset of NHGRI GWAS catalog results with a diverse range of eight selected phenotypes as an example: rheumatoid arthritis, Crohn’s disease, blood pressure, Alzheimer’s disease, breast cancer, pancreatic cancer, colorectal cancer, and prostate cancer. Figure 2 shows a basic PhenoGram plot summarizing the SNP locations for GWA-significant associations with these eight phenotypes. Like the NHGRI GWAS catalog plots, each line connects a chromosomal location to a colored circle depicting the associated phenotype. A key of phenotypes and corresponding circle colors are displayed across the bottom of the image. PhenoGram has multiple options for altering the graphical style of the colored circles. For Figure 2, the options to outline the circles (−O) and increase the phenotype font size (−F) were used.

**Figure 3:**
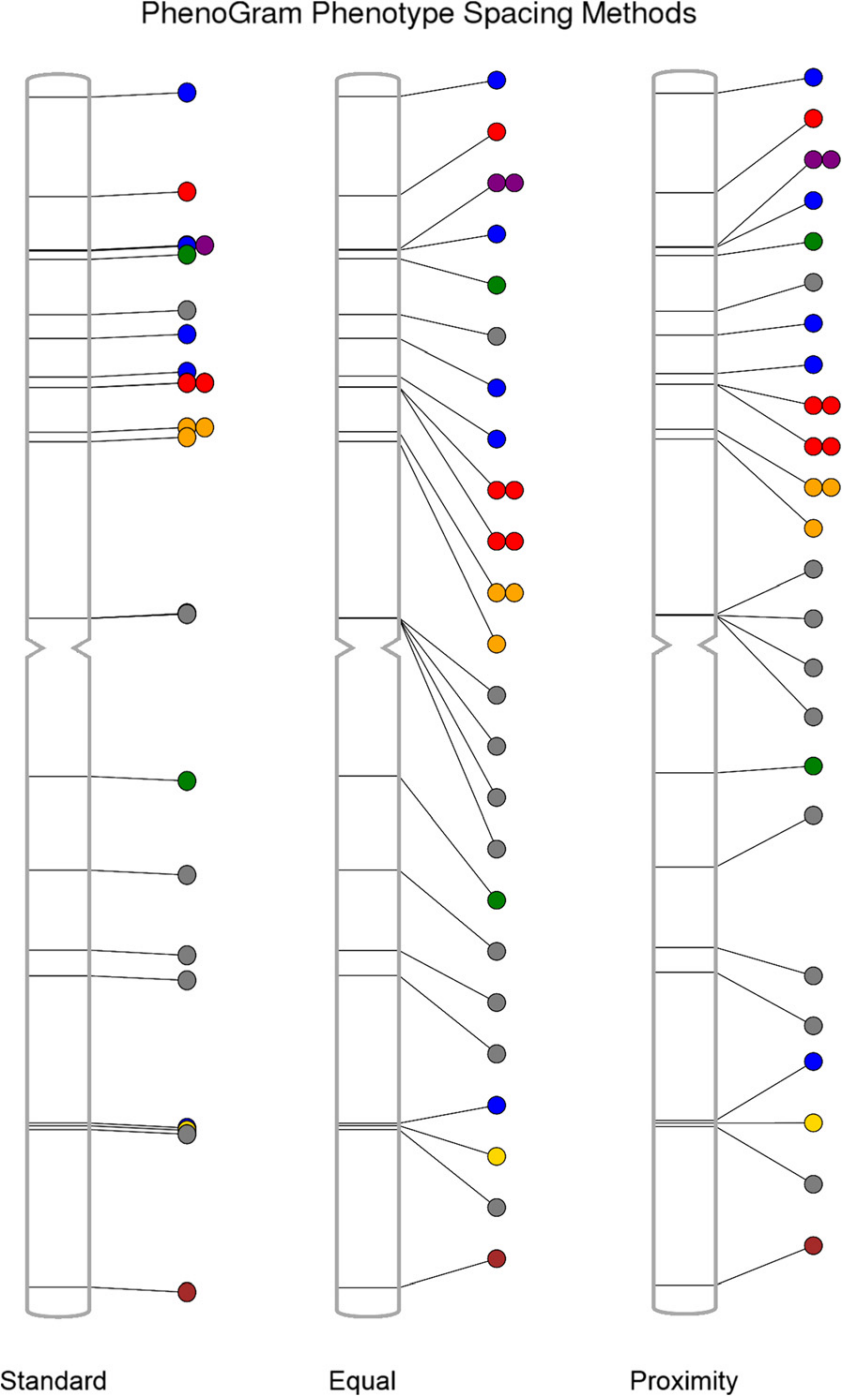
**The different annotation spacing methods available with PhenoGram.** PhenoGram has several options for modifying the spatial presentation of the circles or other annotation on PhenoGram plots: The default of *standard spacing*, the *equal spacing* method placing circles or other annotation at equal intervals along the chromosome, and *proximity spacing* that minimizes circle or annotation overlap while keeping points near their chromosomal locations. The option to plot a single chromosome was used for this figure.

Depending on the amount of data to be plotted, as well as the proximity of genomic regions, different spacing may need to be used to optimally plot multiple data points. For example, an input file with a great number of phenotypes may produce a plot with circles that are too closely juxtaposed. Thus, PhenoGram has several options for modifying the spatial presentation of the circles or other annotation on PhenoGram plots*.* Figure 3 shows the results of using different PhenoGram spacing algorithms that can mitigate the issue of overlapping plotted data. The first spacing method is *standard spacing* and is the default spacing method used by PhenoGram. The *equal spacing* method (−p *equal*) allows the researchers to space the circles at equal intervals along the chromosome. A third spacing method *is proximity spacing* (−p *proximity*) which minimizes circle overlap while still attempting to place circles or other annotation near respective chromosomal locations.

**Figure 4:**
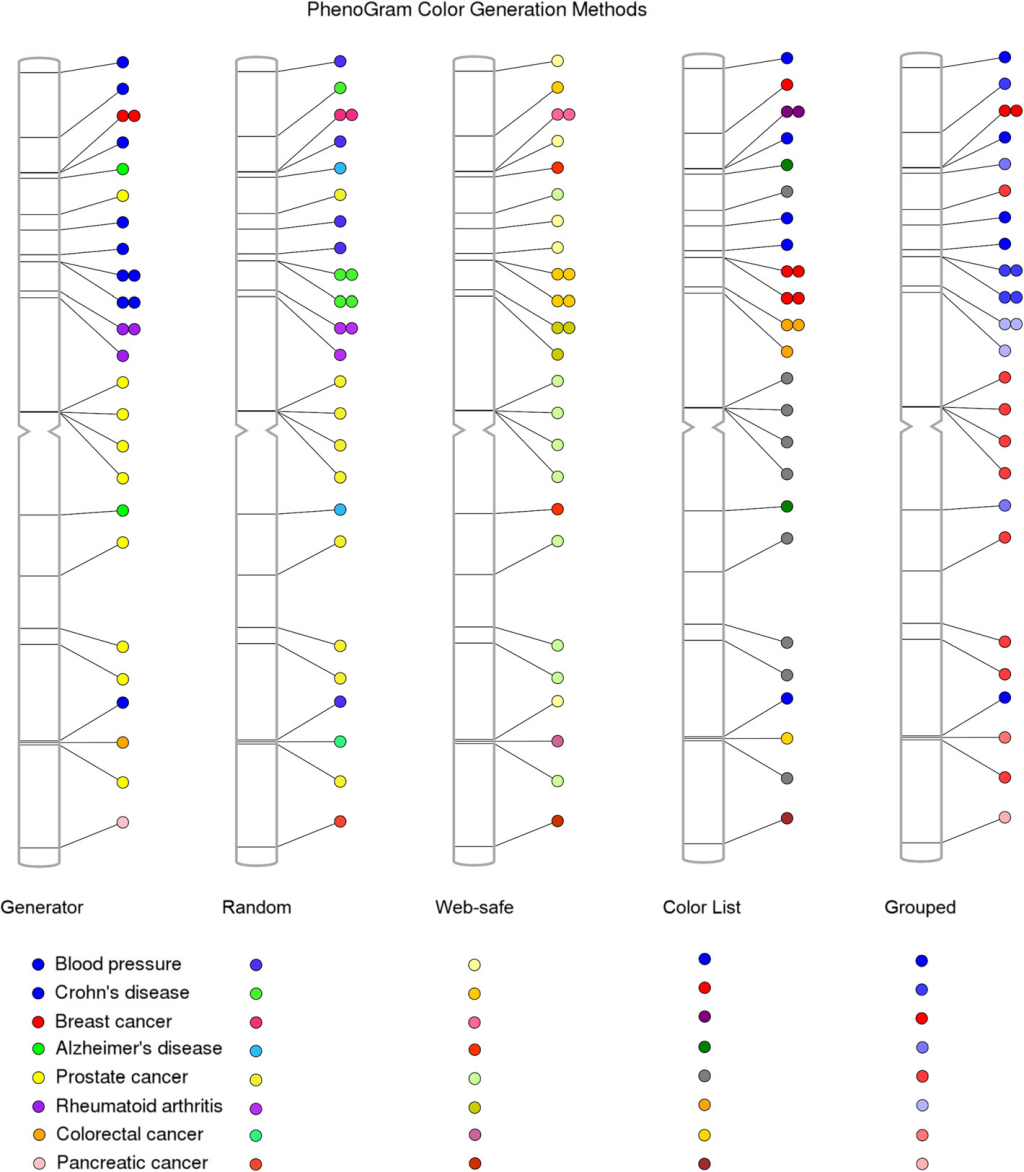
**The five phenotype color generation methods available in PhenoGram.** For a small number of phenotypes, the color list method assigns easily-discernible colors. With a greater number of phenotypes, the standard generator attempts to maximize the color separation between the phenotypes. A random generator may also be used, as well as a method for web-safe colors. The grouped method makes it possible to plot phenotypes with the same designated identifier in a gradient of similar colors.

The colors of the plotted circles can be alternately generated based on five different algorithms, shown in Figure 4. For ten or fewer phenotypes, the color list method (−c *list*) restricts the possible colors to those that are easily differentiated. In plots with a greater number of phenotypes, the standard color generator (−c *generator*) creates colors with maximum separation between all possibilities. The web-safe color option (−c *web*) restricts all possibilities to 216 web-safe, randomly selected possibilities. The least restrictive method is the random generator (−c *random*) that assigns colors without regard for color proximity. Finally, it is possible to provide in the input file a column that designates a group identifier for a subset of phenotypes such that all those of a similar identifier are plotted in a gradient of one color. Figure 4 shows the grouping method (−c *group*) in a plot to differentiate NHGRI GWAS catalog cancer phenotypes from non-cancer phenotypes.

**Figure 5:**
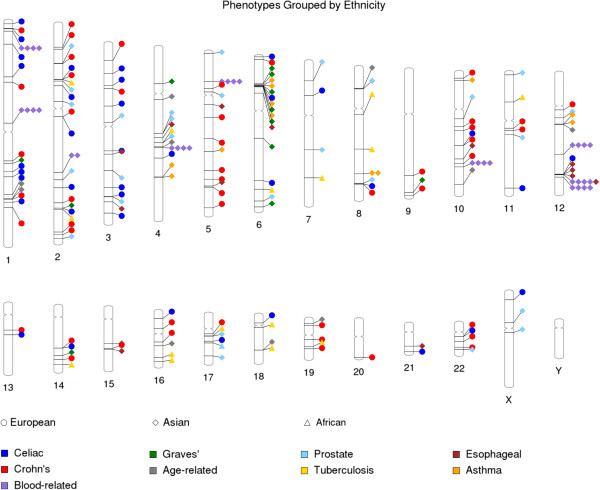
**Adding in a shape to indicate a grouping, such as ancestry.** Designation of ancestry in the PhenoGram input file will result in all phenotypes of each ancestry identifier being plotted with a unique shape in addition to showing a phenotype color. The input file can take up to three ancestry (or other group) identifiers.

Similar to grouping data by phenotype, it is possible to overlay a second grouping by ancestry. Shown in Figure 5, the plot resulting from the incorporation of this data into the input file depicts each ancestry group as a unique shape while still differentiating phenotypes with a color generation method. Here, the phenotype shapes are displayed without a black outline. GWAS catalog data was also used in this plot in order to show the combination of the diverse phenotype colors and distinct shapes by ancestry across the genome. PhenoGram currently accepts up to three different ancestry groups, with each subsequent group beyond three appearing as a circle. Figure 5 displays how PhenoGram can help visualize the relationships between genome location, phenotypes, and ancestry.

**Figure 6:**
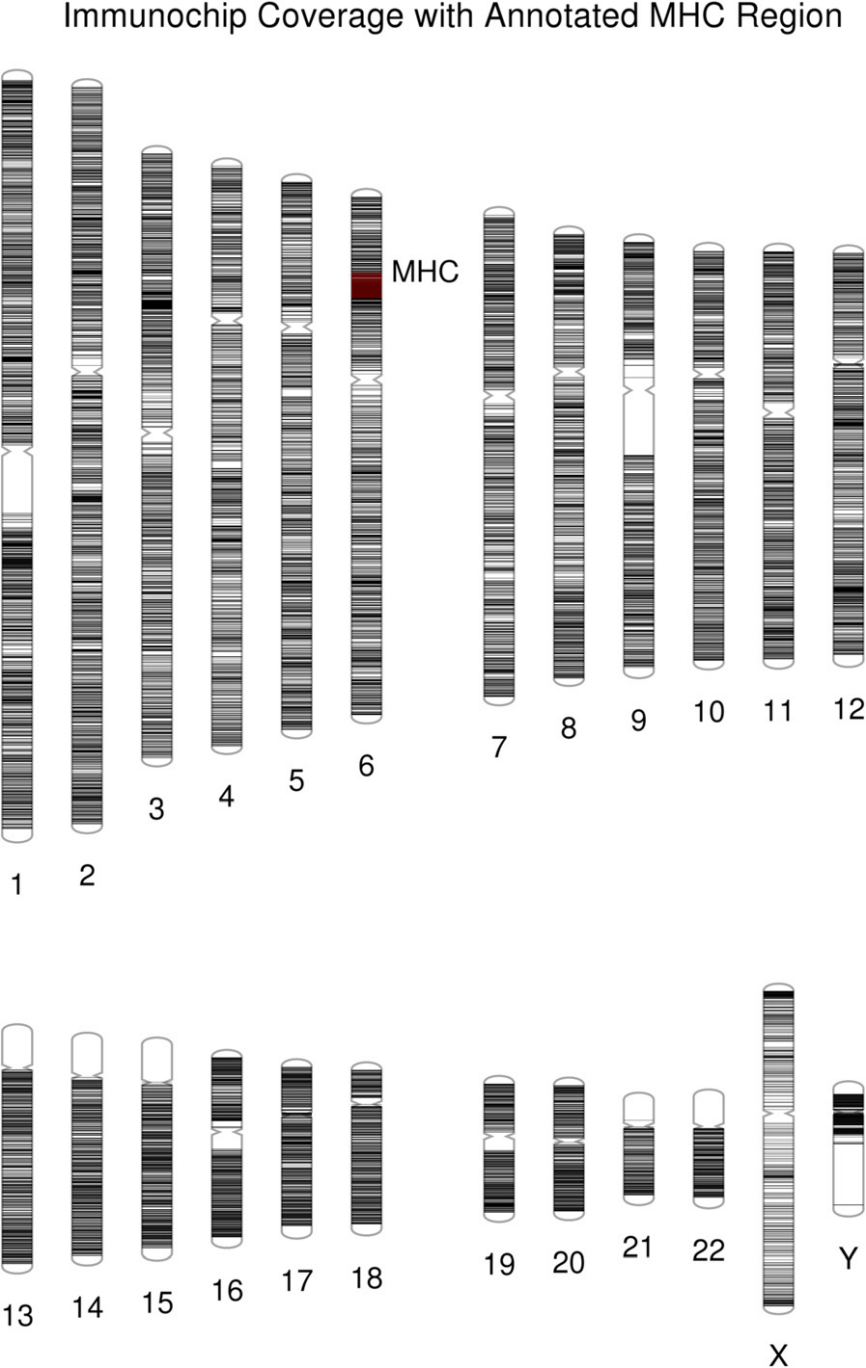
**Plotting lines at base-pair locations using PhenoGram.** Each line represents a base-pair location genotyped on the immunochip genotyping array, an array with variants chosen for previous association with the autoimmune response and the immune system. By setting the lines to be transparent, areas of higher and lower genotyping density are made more visible. The very densely genotyped major histocompatibility region (MHC) on Chromosome 6 has been overplotted with color, as well as annotated, using PhenoGram.

PhenoGram can also create plots that contain, rather than colored shapes, only colored lines that transverse the chromosomes. In this way, the software is also useful for visualizing genome or single-chromosome SNP coverage from a genotyping array as well as to show locations of sequenced loci or other regions of interest. Figure 6 incorporates the line plotting option (−C) with base-pair position information to display the coverage of genotyping for the custom Immunochip genotyping array, an array focused on autoimmune and immune system related genetic variants [6]. Further, it is possible in the PhenoGram input file to highlight base-pair regions via the use of integer-coded color options and to annotate positions. In Figure 6, a dense region of genotyping of the array on chromosome six is annotated; this region is the major histocompatibility complex (MHC) region. In line plots, it may be useful to apply the transparent (−T) or thin (−n) line options to improve visualization in densely plotted genome regions.

**Figure 7:**
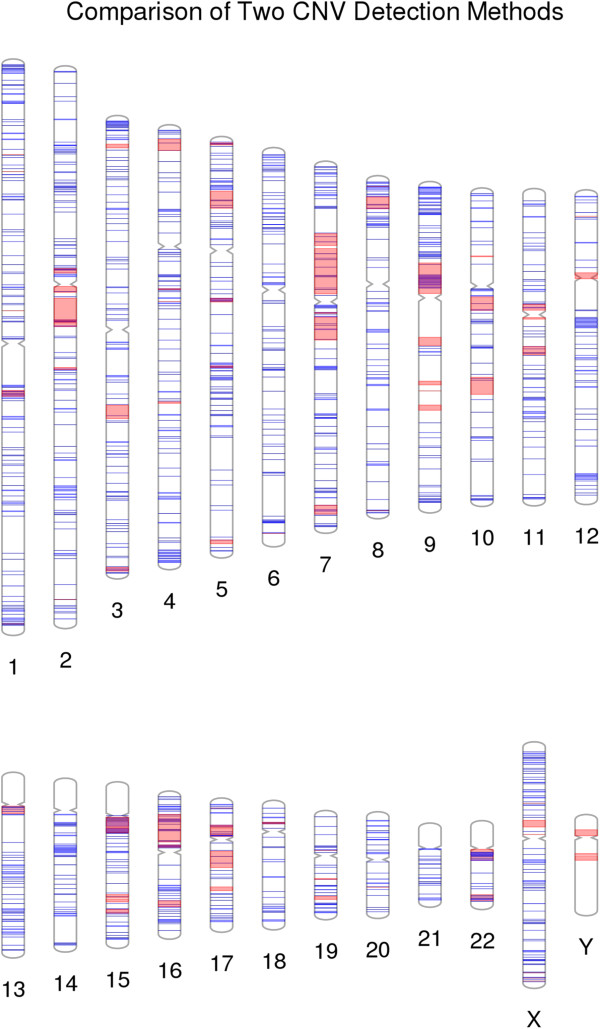
**The CNV detection results of two microarray methods.** Regions of a custom DNA microarray targeted for genomic hotspots, in red, are plotted on top of blue Illumina DNA microarray CNV results.

Copy-number variants (CNVs) are a growing area of genetic variant exploration for neurodevelopmental disorders. Recently, a comparison was made of two microarray technologies used in the detection of CNVs. Figure 7 shows the CNV region overlap between results of an Illumina microarray and a custom microarray that was targeted for genomic hotspots of deletions and duplications [7,8]. Another example, using this approach for single SNPs instead of CNVs (not shown here), would be to use PhenoGram with two different colors highlighting the density and location of a series of low frequency variants vs. the density and location of a series of more common variants.

**Figure 8:**
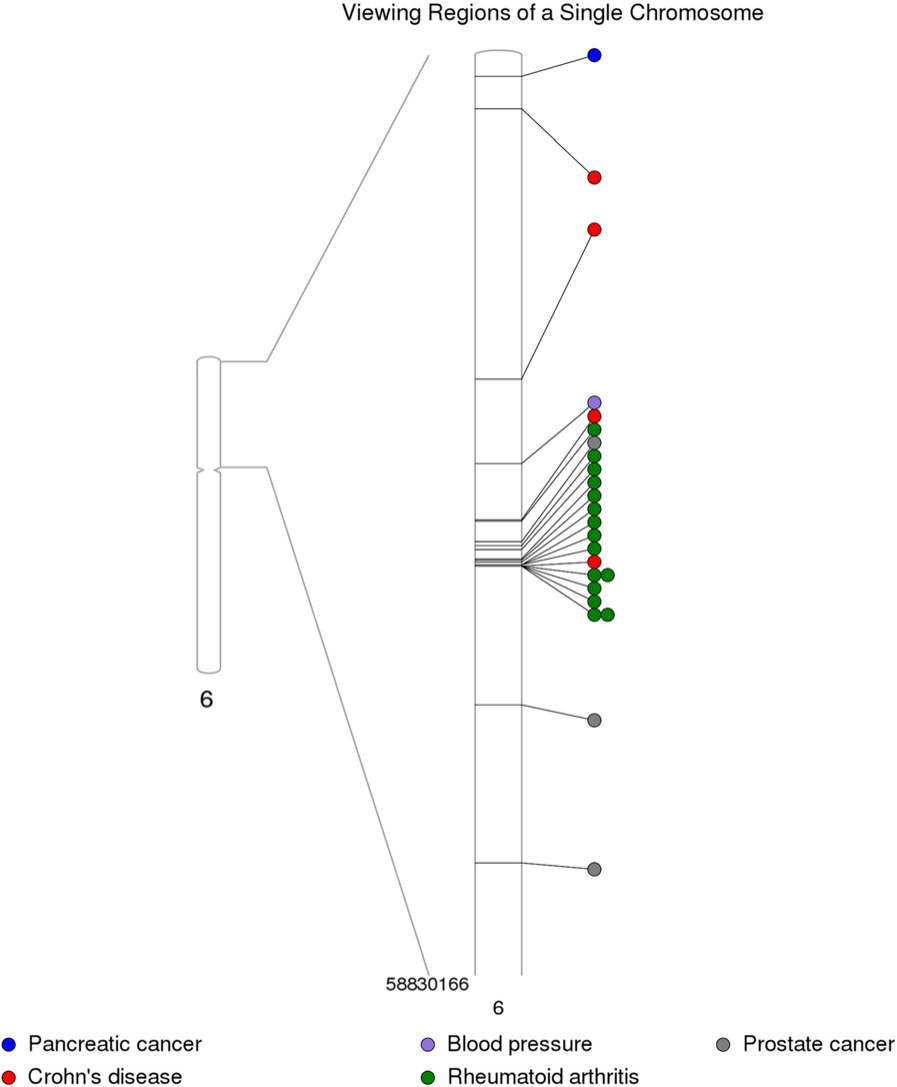
**Using PhenoGram to plot an expanded view of a specific region of a chromosome.** PhenoGram provides the capability of showing a closer view of a specific region of a chromosome. This is particularly useful when there is a heightened density of information to be plotted for a specific chromosomal region. Here, NHGRI GWAS data is shown on a portion of chromosome six where there is a greater density of Crohn’s disease and rheumatoid arthritis phenotypes.

Another option with PhenoGram is to show part of a region in more detail. Depending on the amount of data to be plotted and/or the region of interest, plotting only one chromosome can be useful, and this feature was used to plot individual chromosomes for Figures 3 and 4. Although our annotation spacing algorithms attempt to optimize the presentation of various shapes such as circles representing phenotypes, it can be necessary to visually expand densely annotated chromosomal regions. Figure 8 uses the NHGRI GWAS Catalog data from the eight aforementioned phenotypes to expand on a cluster of closely positioned phenotypes.

**Figure 9:**
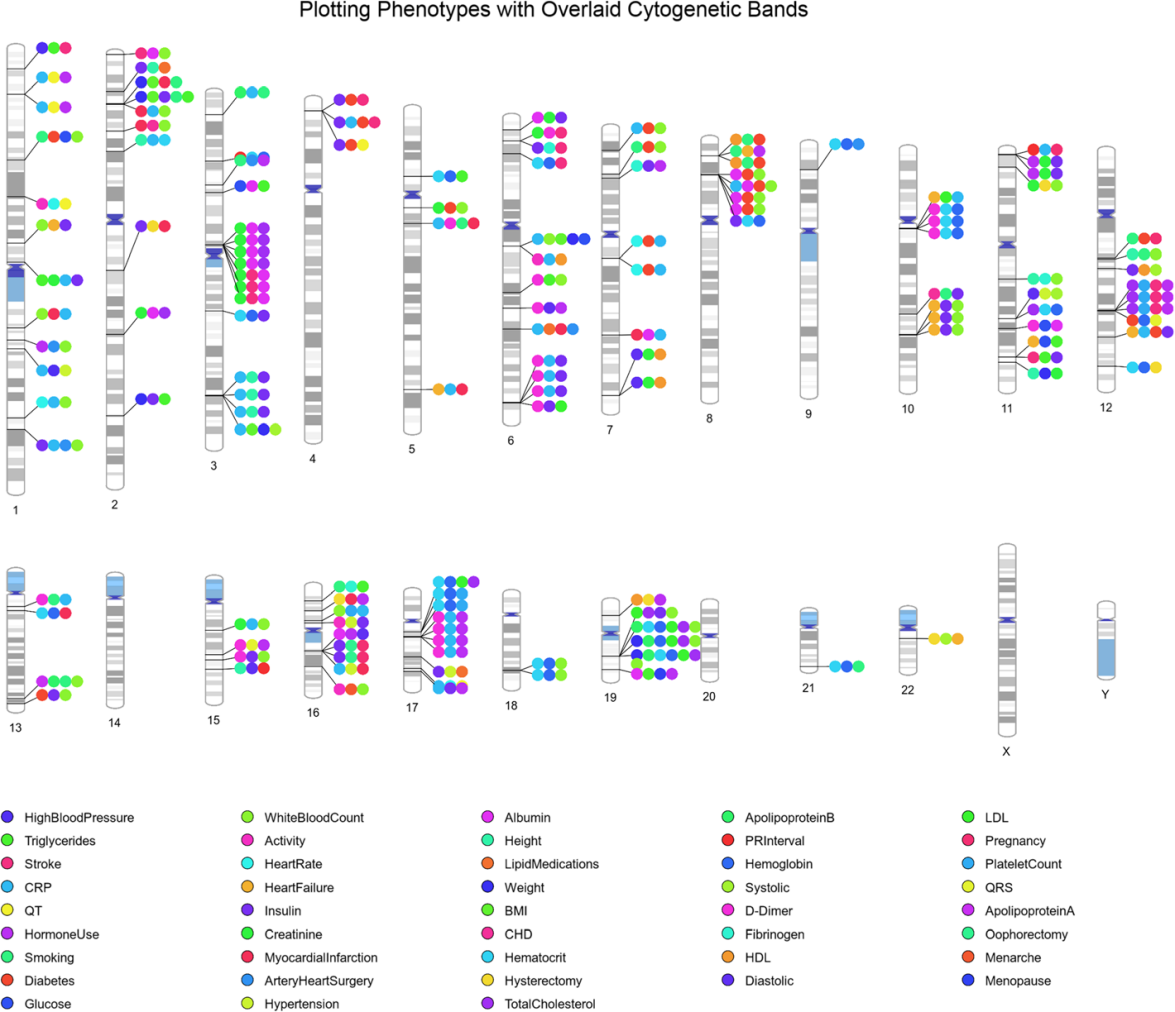
**Cytogenetic band option within PhenoGram.** It is possible to shade the chromosomes with predefined cytogenetic bands on any PhenoGram plot. Here, this option is used with simulated SNP-phenotype association data.

We have added an option in PhenoGram to show the location of cytogenetic bands across the ideogram, and we show an example in Figure 9. Genes are not uniformly distributed along the length of chromosomes. Cytogenetic bands identify biologically relevant chromosomal structure, highlighting regions that are more or less likely to be gene-rich and/or genotyped, and standard regions have been identified that can be visualized on an ideogram documented through the UCSC browser [9] that we downloaded from http://hgdownload.soe.ucsc.edu/goldenPath/hg19/database/. For example, “G-bands” are less gene-rich than “R-bands” [10], and we identify G-bands in PhenoGram using variations of grey and represent R-bands in white on the ideogram. There are also regions of the genome containing highly condensed heterochromatin that are largely transcriptionally silent, we have identified those in dark blue colors. The biggest regions of heterochromatin are in the long arm of the Y-chromosomes and close to the centromeres of chromosomes 1, 9, and 16. Smaller heterochromatin regions are found at the centromere of each chromosome, and the p-arms of chromosomes 13, 14, 15, 21, and 22. We have also marked the “stalks” in light blue, these are five regions on the acrocentric chromosomes and contain genes that code for ribosomal RNA.

## Conclusions

With the ever increasing amounts of data being collected, visually summarizing data can be important for providing insight into complex results. Multiple data results can be plotted across chromosomes, providing useful summary information, and aiding in data analyses as well as sharing results. PhenoGram offers a robust feature set, allowing researchers to plot data of many kinds across a chromosomal ideogram according to preference. In the future we will be adding in additional color option choices for plots, as well as additional software features, to expand plotting options with PhenoGram. The features of PhenoGram can further facilitate the exploration and sharing of genomic information.

### Availability and requirements

Project name: PhenoGram

Project home page: http://visualization.ritchielab.psu.edu

Operating systems(s): Linux, Mac OS X, Windows

Programming language: Ruby

Other requirements: RMagick

License: GNU General Public License

Any restrictions to use by non-academics: PhenoGram use is restricted to academic and non-profit users

## Competing interests

The authors declare they have no competing interests.

## Authors’ contributions

DW, SD, MDR, SAP have made substantial contributions to conception and design of this software, as well as the drafting of the manuscript or revising it critically for important intellectual content, and have given final approval of the version to be published. The writing of the code for PhenoGram was performed by SD. All authors read and approved the final manuscript.
